# Pinocytotic engulfment of lipoproteins by macrophages

**DOI:** 10.3389/fcvm.2022.957897

**Published:** 2022-08-29

**Authors:** Takuro Miyazaki

**Affiliations:** Department of Biochemistry, Showa University School of Medicine, Tokyo, Japan

**Keywords:** CWC22, calpain-6, Rac1, Cdc42, Akt, liver X receptors, macrophage-colony stimulating factor

## Abstract

Atherosclerosis is a major cause of acute coronary syndrome and stroke. Foam cell formation in macrophages is involved in controlling plaque stability and the pathogenesis of atherosclerosis. Accordingly, many studies have examined the processes of lipid incorporation, such as scavenger receptor-mediated uptake of oxidized low-density lipoprotein, in cells. In addition to receptor-mediated machinery, growing evidence has suggested that pinocytosis, which is a receptor-independent endocytic pathway, is associated with foam cell formation when a sufficient number of lipoproteins is accumulated around cells. Pinocytotic engulfment of nanoparticles is initiated by plasma membrane ruffling in a phosphatidylinositol-3 kinase-dependent manner. Subsequent to pinosome closure, the majority of pinosomes are internalized through endocytic processes, and they can be recycled into the plasma membrane. These pinocytotic processes are modulated by small GTPases and their cytoskeletal rearrangement. Moreover, pinocytotic abilities may vary between immunological subsets in cells. Accordingly, macrophages may show diverse pinocytotic abilities depending on the surrounding microenvironment. This review summarizes the current understanding of pinocytotic engulfment of lipoprotein in macrophages, and discusses how this endocytic process is governed under hypercholesterolemic conditions.

## Introduction

Ischemic heart disease is the leading cause of death globally. Atherosclerosis is a major risk factor for coronary disease because expansion of such eccentric thickening of the arterial wall can lead to thromboembolism and thrombotic occlusion. Numerous basic and epidemiological studies have shown that an abnormality of cholesterol handling, excessive adaptive response to vascular insults, and inflammation burden are associated with the pathogenesis of atherosclerosis (1, 2). Among these events, cholesterol accumulation in macrophages is an essential process to expand atherosclerotic plaques and is a major factor for defining plaque stability (3, 4). Several endocytic pathways for internalizing low-density lipoprotein (LDL) cholesterol have been reported. Scavenger receptors, such as scavenger receptor-A, CD36, and lectin-like oxidized LDL receptor 1 (5, 6), in macrophages recognize oxidized LDL, but not native LDL, to internalize LDL through the conventional endocytic process. In addition to the receptor-mediated pathways, macrophages enable the incorporation of nanoparticles through pinocytosis. Pinocytosis is a fundamental cellular process that engulfs extracellular fluids. Pinocytosis is categorized into micropinocytosis and macropinocytosis, which are endocytic processes that engulf small particles (typically < 0.1 μm) and large particles (typically > 0.2 μm), respectively (7). Such pinocytotic engulfment in macrophages, which occurs spontaneously and can be further modified by environmental factors, are related to the innate immune system to monitor surrounding antigens and microbial-associated molecules. Growing evidence has suggested that pinocytosis is mediated through incorporation of native LDL, thereby inducing formation of foamy macrophages (8). This mini review summarizes the recent understanding of internalization and trafficking of pinosomes and their regulatory mechanisms.

## Membrane regulation and endocytic trafficking during pinocytotic incorporation of LDL in macrophages

**Figure 1:**
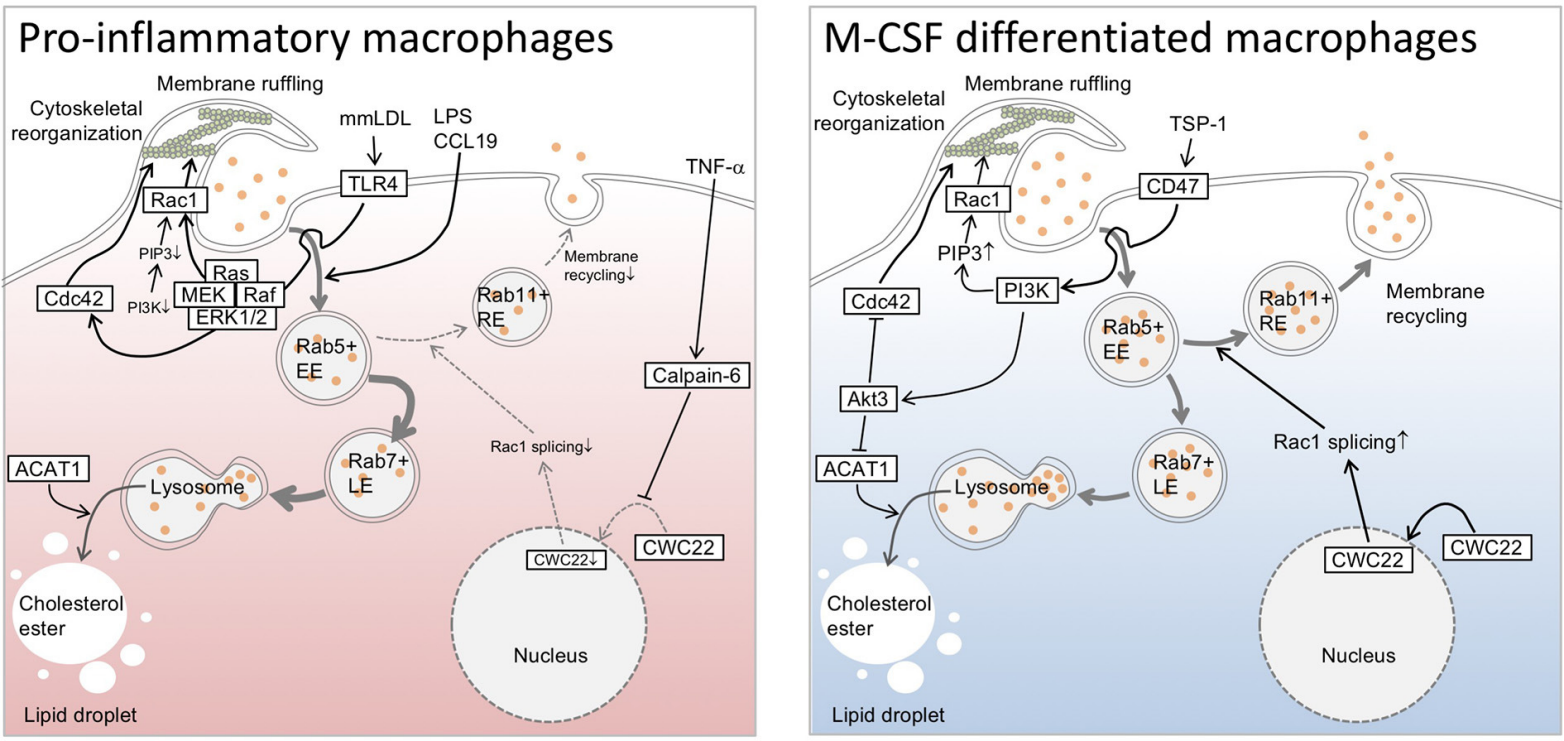
Overview of pinocytotic deposition of low-density lipoprotein cholesterol in macrophages. Phosphoinositide 3-kinase drives pinocytotic plasma membrane ruffling. Small GTPases are associated with trafficking and recycling of pinosomes, as well as with pinocytotic plasma membrane regulation, and have diverse actions in pinocytotic low-density lipoprotein cholesterol uptake in macrophages. While pinocytosis appears to be optimized in an alternative (M2) macrophage subset, certain elements, such as Toll-like receptor 4 (TLR4)-mediated signaling, enable the restoration of pinocytosis, even in an inflammatory (M1) subset. TLR4 driver lipopolysaccharide (LPS) can polarize macrophage differentiation toward M1 subset, and activates pinocytotic activation through unknown mechanisms. Chemokine (C-C motif) ligand 19 reportedly possesses similar pinocytotic effects in the cells. In the case of mmLDL-differentiated macrophages, cytoskeletal rearrangement appears to be driven through TLR4/Ras/Raf/ERK/MEK axis independently of PI3K signaling. Furthermore, pro-inflammatory cytokine TNF-α upregulates calpain-6, a non-proteolytic isoform of calpain protease family, to inhibit CWC22-mediated Rac1 splicing. This interferes with endosomal recycling pathway, and in turn increases lysosomal processing of endosome-derived lipoprotein cholesterol to generate cytosolic lipid droplets. In contrast to M1 subset, macrophage-colony stimulating factor (M-CSF)-differentiated M2 macrophages exhibits phosphatidylinositol 3-kinase (PI3K)-dependent cytoskeletal rearrangement and membrane ruffling. Similarly, thrombospndin-1 enables to elicit CD47-mediated activation of PI3K and subsequent pinocytotic uptake of native LDL. In this case, it is likely that Akt3 negatively regulates Cdc42 and acyl-CoA cholesterol acyltransferase 1 to decelerate pinocytotic cholesterol deposition in the cells. ACAT1, acyl-CoA cholesterol acyltransferase 1; CCL19, Chemokine (C-C motif) ligand 19; EE, early endosome; LE, late endosome; LPS, lipopolysaccharide; M-CSF, macrophage-colony stimulating factor; mmLDL, minimally modified low density lipoprotein; PI3K, phosphatidylinositol 3-kinase; PIP3, phosphatidyl inositol 3-phosphate; Rac1, Rac family small GTPase 1; RE, recycling endosome; TLR4, Toll-like receptor 4; TNF-α, tumor necrosis factor-α; TSP-1, thrombospndin-1.

The pinocytotic deposition of native LDL is sufficient to convert cultured macrophages into foam cells (9). Pinocytotic uptake is independent of the degree of LDL oxidation and does not saturate (7), and the molecular mechanisms underlying pinocytotic LDL uptake are distinct from those of receptor-mediated pathways. Indeed, targeted deletion of the macrophage scavenger receptors scavenger receptor-A and CD36 does not inhibit pinocytotic LDL uptake in macrophage-colony stimulating factor (M-CSF)-differentiated macrophages (10). Generally, macropinocytotic internalization of nanoparticles is divided into plasma membrane ruffling and pinosome closure, which are regulated by submembranous actin organization. Small GTPases have central roles in this cytoskeletal regulation. Ras-GTP drives the activation of phosphoinositide 3-kinases (PI3Ks), which generates patches of PtdIns(3,4,5)P3 (11). The Rho family GTPase Rac and actin-nucleation-promoting factors, such as SCAR/WAVE complex, enable binding to these patches, which nucleates actin filaments. In addition to actin-regulating factors, phospholipase Cγ can be activated by PtdIns(3,4,5)P3, thereby generating diacylglycerol and subsequent activation of protein kinase C, which is involved in the positive regulation of pinocytosis (12). Kruth and colleagues investigated mechanisms underlying pinocytosis using pharmacological approaches in M-CSF-differentiated macrophages, and found that pinocytosis was inhibited by a broad-range PI3K inhibitors (10). In contrast, inhibition of the class I PI3K isoforms *β*, *γ*, or *δ* did not affect micropinocytosis in M-CSF-stimulated macrophages. Similarly, macrophages from mice expressing dominant-negative class I PI3K *β*, *γ*, or *δ* isoforms had no inhibitory effects. Therefore, PI3Ks, excluding class I isoforms, drive macropinocytosis in macrophages. Pharmacological screening of signaling pathways has shown that dynamin, microtubules, actin, and vacuolar type H(+)-ATPase appear to be associated with pinocytotic uptake (10). Accordingly, phosphoinositide metabolism accompanied by cytoskeletal regulation are indispensable even for pinocytotic LDL uptake in macrophages (Figure 1).

In addition to the molecules noted above, the contribution of Rho GTPases to pinocytotic regulation has been well–documented. Anzinger et al. reported that the Rho GTPase inhibitor toxin B substantially inhibited pinocytotic LDL uptake in M-CSF-differentiated human macrophages (13). Using time-lapse microscopy, they found that this inhibitor almost completely inhibited macropinocytosis, although cholesterol deposition in cells was not completely inhibited. Their findings suggest the contribution of another endocytic process, such as micropinocytosis, to LDL cholesterol uptake. In contrast, pharmacological inhibition of Rac1 failed to inhibit pinocytotic LDL uptake in M-CSF-differentiated macrophages (10). Our previous study showed that expression of the Rho GTPases RhoA and Rac1 in bone marrow cells was imperceptible, while it was dramatically induced during the differentiation of cells into macrophages in the presence of M-CSF (14). Treatment of bone marrow cells with tumor necrosis factor (TNF)-α suppressed maturation of *Rac1* mRNA and potentiated macropinocytosis. Deficiency of calpain-6, which is a non-proteolytic isoform of the calpain protease family, in TNF-α-stimulated murine macrophages showed interrupted macropinocytosis concomitantly with the normalization of *Rac1* splicing. Macropinocytosis in calpain-6-deficient macrophages was restored by small interfering RNA-based silencing of *Rac1*. Therefore, aberrant *Rac1* mRNA regulation exerts inhibitory actions in pinocytotic LDL uptake in TNF-α-stimulated macrophages. While the mechanisms by which Rac1 or its splice variants interrupt LDL uptake are unclear, Rac1-mediated regulation of the endosomal recycling pathway may contribute to the inhibitory actions. Indeed, pinosomes in Rac1-expressing macrophages frequently express the recycling endosome marker Rab11. Moreover, macropinosome velocity in cells is decelerated by pharmacological inhibition of Rac1. Therefore, pinocytotic LDL uptake is likely to be due to Rac1-dependent vesicle trafficking rather than Rac1-dependent membrane ruffling. Rho GTPases, such as RhoD, Rac1, Cdc42, TCL, and TC10, are thought to be essential factors for endocytic trafficking (15). Indeed, overexpression of dominant active forms of Rac1 accelerate macropinocytosis activity in rat fibroblasts (16). However, notably, prolonged Rac1 activity impairs the maturation of Rab21-positive pinosomes in macrophages (17), suggesting that optimal Rac1 regulation can maximize pinocytosis. However, Ding et al. showed positive regulation of pinocytotic LDL uptake by Cdc42, which is a small GTPase, in human and murine macrophages (18). Indeed, the loss of Akt3 in murine and human M-CSF-differentiated macrophages upregulates with-no-lysine kinase 1 and subsequently activates serum and glucocorticoid-inducible kinase 1. Serum and glucocorticoid-inducible kinase 1 promotes expression of the Rho family GTPase Cdc42, thereby accelerating cytoskeletal rearrangement and pinocytosis. Similarly, Akt3-dependent negative regulation of pinocytosis is detectable in murine peritoneal macrophages. This regulation is accompanied by limited receptor-dependent uptake of acetylated LDL and downregulation of acyl-CoA cholesterol acyltransferase (19). Acyl-CoA cholesterol acyltransferase converts free cholesterol into cholesterol ester to form cytosolic lipid droplets in peritoneal macropahges (20). Therefore, Akt3 has a protective role in foam cell formation in macrophages and atherogenesis. How Akt3 simultaneously downregulates receptor-dependent and receptor-independent pathways is currently unclear, but Akt3 might interfere with a common signaling pathway, such as endocytic vesicle trafficking or the exocytotic process. This possibility is consistent with a previous study, which showed that overexpression of dominant-negative Cdc42 counteracted macropinocytosis in vascular endothelial cells (21). Collectively, small GTPases are associated with vesicle trafficking and pinocytotic plasma membrane regulation (Figure 1), and have diverse actions regarding pinocytotic LDL uptake in macrophages. Therefore, defining pinocytotic activity by expression levels of small GTPases alone is difficult.

## Regulatory mechanisms underlying pinocytotic LDL incorporation

Macrophages can be divided into a variety of subpopulations, which comprise pro-inflammatory, immunosuppressive, and tissue-repairing types, and they are dynamically interconverted depending on the tissue microenvironment (22). During bacterial infection, monocyte-macrophages can be activated by inflammatory elements, such as Toll-like receptor (TLR) ligands and interferon-γ, which facilitate the skewing of macrophages into the M1 subset (classically activated macrophages). M1 macrophages produce inflammatory cytokines, such as TNF-α, interleukin (IL)-6, and IL-12, reactive oxygen species, and reactive nitrogen species, and induce a Th1-type immune response (23). Furthermore, M1 macrophages exert strong antibacterial or antiviral activity and antitumor effects. In contrast to the M1 subset, macrophages activated by IL-4 and IL-13 produced by Th2 cells, basophils, mast cells, and innate lymphoid cells are converted into M2 macrophages (alternatively activated macrophages) (23). This activation exerts host defense against parasites, tissue repair, angiogenesis, and tumor growth, and becomes immunosuppressive. M2 macrophages strongly express arginase and mannose receptors (24). Tumor-associated macrophages that are infiltrated into tumor tissue are thought to be converted from M1 to M2 subsets, thereby accelerating tumor progression (25). Tumor-associated macrophages showing low IL-12 expression levels and high IL-10 expression levels, possess weak antitumor activity, and drive matrix remodeling and angiogenesis (26). In addition to the progression of cancer, aberrant regulation of macrophage subsets can be involved in various diseases. Therefore, phenotypic regulation of macrophage subsets can be regarded as a therapeutic target. Arteriosclerosis, which is a Th1-dominant vascular disorder, can be treated by skewing M1 to M2 macrophages (27). In contrast, bronchial asthma, which is a Th2-dominant disease, might be targeted by the opposite strategy (28).

M2 macrophages show constitutive pinocytotic activity and can be further stimulated with related cytokines such as M-CSF. Notably, the majority of the above-mentioned observations on pinocytotic LDL uptake were from investigations using M-CSF-differentiated human monocyte-derived macrophages. Redka et al. showed that M-CSF/IL-4-differentiated M2 macrophages had robust micropinocytosis activity, while that in granulocyte M-CSF/interferon-γ/lipopolysaccharide-differentiated M1 macrophages was negligible (29). They found that Rho GTPase expression levels in M1 macrophages were lower than those in the M2 subset. In addition to small GTPases, insufficient PI3K activity is likely responsible for modest pinocytotic ability in the M1 subset (29). Calcium-sensing receptors appear to be necessary for sustaining small GTPases and PI3K in M2 macrophages. However, notably, M1 macrophages show robust pinocytotic activity when the cells are stimulated with certain pro-inflammatory substances. Indeed, acute treatment of pro-inflammatory macrophages with lipopolysaccharide or CC chemokine ligand 19 markedly accelerates macropinosome formation in human monocyte-derived macrophages (29). Moreover, minimally oxidized low-density lipoprotein (mmLDL), which is an alternative TLR4 ligand, and cholesteryl ester hydroperoxide, an active component of mmLDL, induce TLR4-dependent macropinocytotic incorporation of oxidized LDL and native LDL (30). In this case, mmLDL elicits an association of spleen tyrosine kinase with a TLR4 complex, TLR4 phosphorylation, activation of the Vav1-Ras-Raf-MEK-ERK1/2 axis, phosphorylation of paxillin, and activation of Rac, Cdc42, and Rho in murine peritoneal macrophages. These findings suggest that mmLDL-induced TLR4 signaling normalizes the disparity of small GTPases. Collectively, the status of small GTPases is likely to depend on types of extracellular stimuli rather than synchronizing with the phenotypic status of M1/M2 subsets. Accordingly, the master regulator of pinocytosis is currently unclear.

While the regulatory mechanisms underlying pinocytosis are poorly understood, several studies have focused on the upstream modulator of this process (Figure 1). Agonists of liver X receptors (LXRs) are involved in the downregulation of pinocytotic uptake of native LDL in M-CSF-differentiated macrophages (31). LXRs are ligand-activated transcription factors involved in the control of lipid metabolism and inflammation. Because targeting LXRs in bone marrow cells facilitates atherosclerosis (32), LXRs may interfere with pinocytotic cholesterol deposition in macrophages and subsequent pathogenesis of atherosclerosis. Csányi et al. found that thrombospndin-1 (TSP-1) and its cytoskeletal regulation potentiated pinocytosis (33). Indeed, treatment of TSP-1 with human and murine M-CSF-differentiated macrophages stimulated membrane ruffle formation and pericellular solute internalization by macropinocytosis. The TSP1 cognate receptor CD47, NADPH oxidase 1 (Nox1) signaling, PI3K, and myotubularin-related protein 6 appear to be associated with TSP1-induced macropinocytosis. Our previous study showed that CWC22, which is an essential loading factor of exon junction complex, was associated with pinocytotic incorporation of native LDL in murine bone marrow-derived macrophages (14). CWC22 in macrophages shows nuclear localization in human mild atherosclerotic lesions, while it shows cytosolic localization in advanced atherosclerotic lesions. Macrophages in advanced lesions simultaneously express calpain-6, which potentiates formation of a calpain-6/CWC22 complex in the cytoplasm and inhibits nuclear localization of CWC22. Because CWC22 has a direct role in *Rac1* mRNA splicing, calpain-6 counteracts CWC22-mediated maturation of *Rac1* mRNA. Indeed, calpain-6-deficient macrophages show low Rac1 protein expression levels and insufficient macropinocytosis activity. Knockdown of *Cwc22* in calpain-6-deficient macrophages leads to the recovery macropinocytosis, concomitantly with restoring *Rac1* mRNA maturation. As noted above, Rac1 facilitates the dynamics of recycling endosomes. Therefore, CWC22 and related splicing factors are thought to be negative regulators of the pinocytotic process.

## Does pinocytosis drive atherogenesis?

While fluid phase pinocytosis is a constitutive process in macrophages and other cells under the physiological conditions, this process is reportedly potentiated in former cell type when they are localized in atherosclerotic lesions. *In vivo* experiments using *Apoe*^−/−^ hypercholesterolemic mice have indicated that fluorescent nanobeads, which is similar in size to LDL and were injected into blood circulation, were massively accumulated in CD68-positive macrophages in atherosclerotic lesions, but not in non-atheroma regions in arteries (34). We also investigated the uptake of nanobeads using *Ldlr*^−/−^ hypercholesterolemic mice. As a result, similar deposition of nanoparticle was reproduced in foamy macrophages in atherosclerotic lesions (14). These observations suggest that macrophages in atherosclerotic lesions preferentially engulf environmental LDL-like particles under hypercholesterolemia. Interestingly, targeting calpain-6 suppressed nanobeads incorporation in foamy macrophages as well as atherogenesis in hypercholesterolemic mice without altering plasma lipid profiles. Notably, overexpression of calpain-6 can upregulate pinocytotic incorporation of LDL in bone marrow-derived macrophages without modifying receptor-mediated uptake of oxidative LDL and phagocytic uptake of aggregated LDL. Considering that calpain-6 is exclusively expressed in macrophages in atherosclerotic lesions, it is interpreted that the pinocytotic incorporation of LDL in lesional macrophages, at least of their calpain-6-mediated portion, may be responsible for the pathogenesis of atherosclerosis. Nevertheless, since calpain-6 contribute to the other atherogenic processes in macrophages such as cellular motility (14), the pathophysiological importance of this endocytic process in the pathogenesis of atherosclerosis has not been fully determined.

## Discussion

As noted above, the contribution of pinocytosis to the pathogenesis of atherosclerosis is currently sketchy. This is because of the lack of responsible regulatory element(s) of this processes. Cell-based experiments suggest the dominant roles of small GTPases in pinocytotic membrane ruffling, while they also contribute to the other fundamental processes of cells, including mitosis and cell motility. Identification of the pinocytotic master regulator enables to perform intervention study in animal models to determine the pathogenic significance of this processes. It was reported that targeted deletion of scavenger receptors, CD36, and scavenger receptor-A in hypercholesterolemic mice inhibits the pathogenesis of atherosclerosis without altering oxidized LDL uptake in macrophages (35). Therefore, pinocytotic uptake of LDL in the cells is worthy to be further investigated. To identify the master regulator of pinocytotic LDL uptake, comprehensive studies evaluating membrane regulation, vesicle trafficking, exocytosis/efflux, and subsequent cholesterol deposition are necessary.

## Author contributions

TM conceived and designed the review, appraised the literature, and wrote the manuscript.

## Funding

This study was supported in part by the Japan Society for the Promotion of Science KAKENHI grants [grant nos.: JP19K08590 and JP22H03520 (to TM)], a research grant from Bristol-Myers Squibb (to TM), a research grant from the Mochida Memorial Foundation for Medical and Pharmaceutical Research (to TM), and a research grant from the Naito Memorial Foundation (to TM).

## Conflict of interest

The author declares that the research was conducted in the absence of any commercial or financial relationships that could be construed as a potential conflict of interest.

## Publisher's note

All claims expressed in this article are solely those of the authors and do not necessarily represent those of their affiliated organizations, or those of the publisher, the editors and the reviewers. Any product that may be evaluated in this article, or claim that may be made by its manufacturer, is not guaranteed or endorsed by the publisher.
